# The Signature Sequence Region of the Human Drug Transporter Organic Anion Transporting Polypeptide 1B1 Is Important for Protein Surface Expression

**DOI:** 10.1155/2014/129849

**Published:** 2014-10-09

**Authors:** Jennina Taylor-Wells, David Meredith

**Affiliations:** Department of Biological & Medical Sciences, Faculty of Health & Life Sciences, Oxford Brookes University, Gipsy Lane, Headington, Oxford OX3 0BP, UK

## Abstract

The organic anion transporting polypeptides (OATPs) encompass a family of membrane transport proteins responsible for the uptake of xenobiotic compounds. Human organic anion transporting polypeptide 1B1 (OATP1B1) mediates the uptake of clinically relevant compounds such as statins and chemotherapeutic agents into hepatocytes, playing an important role in drug delivery and detoxification. The OATPs have a putative 12-transmembrane domain topology and a highly conserved signature sequence (human OATP1B1: DSRWVGAWWLNFL), spanning the extracellular loop 3/TM6 boundary. The presence of three conserved tryptophan residues at the TM interface suggests a structural role for the sequence. This was investigated by site-directed mutagenesis of selected amino acids within the sequence D251E, W254F, W258/259F, and N261A. Transport was measured using the substrate estrone-3-sulfate and surface expression detected by luminometry and confocal microscopy, facilitated by an extracellular FLAG epitope. Uptake of estrone-3-sulfate and the surface expression of D251E, W254F, and W258/259F were both significantly reduced from the wild type OATP1B1-FLAG in transfected HEK293T cells. Confocal microscopy revealed that protein was produced but was retained intracellularly. The uptake and expression of N261A were not significantly different. The reduction in surface expression and intracellular protein retention indicates a structural and/or membrane localization role for these signature sequence residues in the human drug transporter OATP1B1.

## 1. Introduction

The organic anion transporting polypeptides (OATPs) are a family of membrane transport proteins capable of transporting an array of structurally diverse endogenous and xenobiotic compounds [1–3]. The proteins are expressed in a variety of tissues, including absorptive/excretory cells of the liver and kidney, acting as both a drug delivery and a drug detoxification system.

The human organic anion transporting polypeptide 1B1 (OATP1B1) (gene symbol* SLCO1B1*, previously OATP2, OATP-C, and LST-1) [4] is expressed solely on the basolateral membrane of hepatocytes, facilitating the uptake of clinically relevant compounds such as antibacterial drugs, statins, and chemotherapeutic drugs [5]. As well as playing an important role in hepatic drug disposition [6], OATP1B1 also acts as an active drug delivery system for statins to the liver as a target organ [7]. The protein has been implicated in many drug-drug interactions, including those with cyclosporine A, rifampicin, and statins [8–10]. Many individual DNA nucleotide changes known as single nucleotide polymorphisms (SNPs) have also been located in OATP1B1, contributing to changes in the absorption, distribution, metabolism, and excretion (ADME) of compounds [5, 11]. Finally, studies have shown that in tumour tissues the normal liver specific OATP1B1 is highly expressed [12, 13], suggesting that this transporter may be a suitable target for tumour immunotherapy [12]. Therefore knowledge of the structure and function of this transporter is imperative to understand how the delivery of xenobiotic compounds occurs.

OATP1B1 has been previously predicted by topology prediction analyses to contain 12 transmembrane domains (TMs) and intracellular amino (N) and carboxyl (C) termini [2, 14]. Meier-Abt et al. modeled OATP1B3 on the major facilitator superfamily transporters, glycerol-3-phosphate (GlpT) and lactose permease [15], also predicting the 12-TM topology. Experimental analyses with epitope tagging have inferred that rat OATP1A1 has 12 TMs and confirmed that the C-terminus of OATP1B1 is located intracellularly [16, 17].

The OATPs are also characterized by a conserved 13 amino acid signature sequence (D-X-RW-(I,V)-GAWW-XG-(F,L)-L) (Table 1) at the border between the extracellular loop 3 and TM 6. The position of this sequence at the TM interface, combined with the presence of three highly conserved tryptophan residues, suggests a potential role in stabilizing the protein within the membrane [19, 20]. To date, only the portion of the sequence predicted to encompass TM6 has been studied (A257, W258, W259, L260, N261, F262, and L263) [21, 22]. Mutation of W258, W259, and F262 to alanine in OATP1B1 showed a significant decrease in transport, suggesting a role in protein function [21]. The remaining mutants were unaffected.

Tryptophan could be an important amino acid for determining the structure of a transmembrane protein owing to its nonpolar surface area, the capability for hydrogen bond formation, and a high electrostatic potential [23]. Individual mutation of tryptophans W258 and W259 in human OATP1B1 to alanine however had differing effects on the transport of different substrates, but surface expression was unaffected [21]. In contrast, when the equivalent tryptophans were mutated individually and as a double mutant to phenylalanine in rat OATP1C1, protein function was preserved [21, 22].

In this study, the topology of OATP1B1 was predicted using homology modelling software and the consensus of 10 topology prediction programs. These results were used to predict the position of the TMs and the signature sequence. The programs predicted a 12-TM protein with the signature sequence spanning the extracellular loop 3 and TM 6. These results were then used to place a FLAG epitope into the extracellular loop 2 of OATP1B1, for the detection of protein on the membrane.

The function of the OATP1B1 signature sequence was investigated by site-directed mutagenesis of the most conserved amino acids within the sequence D251, W254, W258/W259, and N261A (Table 1). This included those predicted within extracellular loop 3, D251, and W254, which have not been previously studied. Conservative mutations were made to the chosen amino acids to assess the importance of the amino acid for protein function. Therefore, the polar aspartic acid was mutated to glutamic acid (D251E), both being negatively charged polar residues. The nonpolar tryptophans W254 and W258/259 were mutated to the structurally similar and aromatic phenylalanine (W254F and W258/259F, resp.). In addition, asparagine 261 (N261) in the OATP1B1 sequence is not conserved among the vast majority of the other OATPs, where this residue is a nonpolar glycine. Therefore N261 was mutated to alanine to investigate the role of this amino acid in the sequence.

Transport function by OATP1B1 was measured using the radiolabelled substrate [^3^H]estrone-3-sulfate ([^3^H]E3S) and surface expression was detected via the FLAG epitope in HEK293T cells using luminometry, a quantitative chemiluminescence method, and confocal microscopy following immunofluorescence. Results revealed a significant reduction in surface expression and transport for the D251E, W254F, and W258/259F signature sequence mutants, with confocal microscopy revealing the protein to be retained intracellularly. N261A transport and surface expression were comparable to the wild type.

## 2. Materials and Methods

### 2.1. Materials

Radiolabeled [^3^H]estrone-3-sulfate ([^3^H]E3S) (54.3 Ci/mmol) was purchased from Perkin Elmer (MA, USA). All other chemicals including the anti-FLAG antibodies were purchased from Sigma Aldrich (Dorset, UK), unless stated otherwise.

### 2.2. Topology Prediction

The topology of OATP1B1 was predicted using the online prediction programs TOPCONS [24], Phobius [25], TMpred [26], TopPred II [27], Membrain [28], PHDhtm [29], Split 4.0 [30], Philius [31], HMMTOP [32], SVMtm [33], ConPred II [34], SOSUI [35], TMHMM [36], TSeg [37], PRED-TMR [38], and MEMSAT3 [39]. A homology model was generated using the Protein Homology/analogY Recognition Engine V 2.0 (PHYRE2) [40], which modeled the OATP1B1 sequence on the known structure of GlpT [41].

### 2.3. Cloning and Mutagenesis

The* SLCO1B1* gene was cloned into the mammalian vector pcDNA3.1/Hygro(-) (Life Technologies Ltd., Paisley, UK) with* XhoI* and* KpnI*. An overlap extension PCR method [42] was used to insert the 24-base DNA sequence for the FLAG epitope (DYKDDDDK) into the putative extracellular loop between TMs 3 and 4 (termed OATP1B1-FLAG). The signature sequence mutations were made using overlap extension site-directed mutagenesis of the extracellularly tagged OATP1B1-FLAG construct, which was then used as the wild type. All cloning and mutagenesis were confirmed by DNA sequencing. FLAG primers were as follows: OATP1B1 N-term, 5′-ttttttctcgaggccaccatggaccaaaatcaac-3′; OATP1B1 C-term, 3′-aaaaaaggtacctcccttaacaatgtgtttc-5′; OATP1B1-FLAG, 5′-caacatcaaccttatccactgattataaggacgacgacgacaagtgtttaattaatcaaattttatc-3′ (complementary). Complementary forward primers for the signature sequence mutants were as follows: D251E, 5′-ggataactcctactgagtctcgatgggttgg-3′; W254F, 5′-ctactgattctaaatttgttggagcttggtgg-3′; W258/259F, 5′-cgatgggttggagctttcttccttaatttccttgtg-3′; N261A, 5′-ttggagcttggtggcttgctttccttgtgtctggactattctc-3′.

### 2.4. Protein Expression in HEK293T Cells

Human embryonic kidney 293T (HEK293T (ATCC, Manassas, VA)) cells were grown at 37°C with 5% CO_2_ in Dulbecco's modified eagles medium, supplemented with 10% FBS (Life Technologies, Paisley, UK), 1X penicillin-streptomycin, 250 *μ*g/mL amphotericin-B, 2 mM L-glutamine, and 1X nonessential amino acids solution. Cells at ~80% confluence were harvested using 1X accutase solution (Millipore, Watford, UK) and transfected with the Amaxa Cell Line Nucleofector kit V using a Nucleofector II device (Lonza, Preston, UK) as per the manufacturer's instructions, before plating at 1.6 × 10^4^ cells/well in 12-well plates for transport assays and immunofluorescence or 3.3 × 10^4^ cells per 35 mm dish for luminometry. Plates and dishes were precoated with 0.1 mg/mL poly-D-lysine.

### 2.5. [^3^H]E3S Transport Assay

Cells in 12-well plates at 72 hours after transfection were removed from the incubator and the media were aspirated. Wells were washed three times with 300 *μ*L uptake solution (142 mM NaCl, 5 mM KCl, 1 mM KH_2_PO_4_, 1.2 mM MgSO_4_, 1.5 mM CaCl_2_, 5 mM glucose, and 12.5 mM HEPES to pH 7.3), prewarmed to 37°C. Cells were then incubated in 300 *μ*L 0.1 *μ*M [^3^H]E3S plus 0–1.5 *μ*M unlabeled substrate in uptake solution for 3 min at 37°C. Transport was stopped by the addition of 300 *μ*L ice cold uptake solution. Each well was washed a further three times with ice cold uptake solution before the addition of 200 *μ*L 1% SDS/0.5 M NaOH and shaken overnight at room temperature to lyse the cells. For quantification 50 *μ*L cell/SDS/NaOH solution was removed and 200 *μ*L Optiphase Supermix scintillation cocktail (Perkin Elmer, Witney UK) was added before measuring in a Perkin Elmer 1450 Microbeta Trilux liquid scintillation counter. The remaining solution was used to quantify protein levels using the BCA protein assay kit (Merck, Feltham UK). The [^3^H]E3S uptake was normalized to the protein content in each well, to give transport in pmol/mg protein/3 min.

### 2.6. Kinetic Analysis

Kinetic parameters for transport of [^3^H]E3S by OATP1B1 were determined under initial rate conditions. Transport of [^3^H]E3S was measured in the presence of unlabeled E3S (0–1.5 *μ*M) and mediated transport calculated by subtracting the uptake seen after transfection with the empty vector. The Michaelis-Menten equation was applied to obtain estimates of the maximal uptake rate (*V*_max⁡_) and the apparent affinity constant (*K*_*m*_).

### 2.7. Luminometry Assay

The luminometry assay was adapted from the method described by Paavola et al. [43]. Cells in 35 mm dishes at 72 hours after transfection were washed and fixed with 1 mL 4% paraformaldehyde. Cells were then blocked with 1 mL 2% dry nonfat milk in PBS for 30 min, before incubating with 1 mL HRP-conjugated anti-FLAG antibody (1 : 1000 in milk buffer) for 2 hours at room temperature. Dishes were washed twice with milk buffer then once with PBS before adding substrate (Pierce, Loughborough, UK) and analysing in a TD-20/20 luminometer (Turner Designs, Alton, UK). Luminometry readings were taken with a 5-second delay and 30-second measurement. The cells were lysed with 1 mL of 1% SDS/0.5 M NaOH and shaken overnight at room temperature. Protein was quantified using the BCA protein assay kit (Merck, Feltham, UK) to allow normalization of the luminescence values (relative light units (RLU) to the protein content (RLU/mg protein) in each dish.

### 2.8. Immunofluorescence and Confocal Microscopy

HEK293T cells were grown on 15 mm cover slips in 12-well plates for 48 hours after transfection. Wells were washed and cells were fixed by the addition of 4% paraformaldehyde, 4% sucrose for 15 minutes. Wells were washed with PBS three times for 5 minutes per wash. After blocking with 10% BSA in PBS for 30 minutes at 37°C, cells were incubated with primary monoclonal mouse anti-FLAG antibody (1 : 2000 in 5% BSA/PBS) for 2 hours at 37°C. Wells were washed with PBS three times/5 min, followed by incubation with anti-mouse IgG-fluorescein isothiocyanate (FITC) rabbit secondary antibody (1 : 1000 in 5% BSA/PBS) for 45 minutes at 37°C. Wells were washed with PBS three times/5 min. Before mounting, wells were incubated with 1 *μ*g/mL 4′,6-diamidino-2-phenylindole (DAPI) for 2 minutes. Cover slips were mounted face down over mounting media containing 1,4-diazabicyclo[2.2.2] octane (DABCO) on a standard glass slide. Slides were visualized on the x40 lens of a Zeiss LSM 510 META confocal microscope. Images were acquired with fixed settings to allow comparison between samples.

### 2.9. Statistical Analysis

Statistical significance of data was calculated using a two-tailed unpaired Student's *t*-test (Graphpad Prism 5, CA USA) and statistical significance of normalized data was calculated using a two-tailed one sample *t*-test (Sigmaplot 11.0, Systat Software, CA, USA), both with *P* < 0.05 as significant.

## 3. Results

### 3.1. OATP1B1 Topology Results

A screen of all available topology prediction programs was made to accurately predict the topology of OATP1B1, to allow identification of an insertion site for the FLAG eiptope. 16 programs were evaluated, 10 of which predicted OATP1B1 to contain 12 TMs with intracellular N and C termini (TOPCONS, Phobius, TMpred, TopPred II, Membrain, PHDhtm, Split 4.0, Philius, HMMTOP, and SVMtm). The remaining 6 programs predicted 11 TMs (ConPred II, SOSUI, TMHMM, TSeg, PRED-TMR, and MEMSAT3), with TM4 from the 12-TM model not present in the 11-TM model.

**Figure 1:**
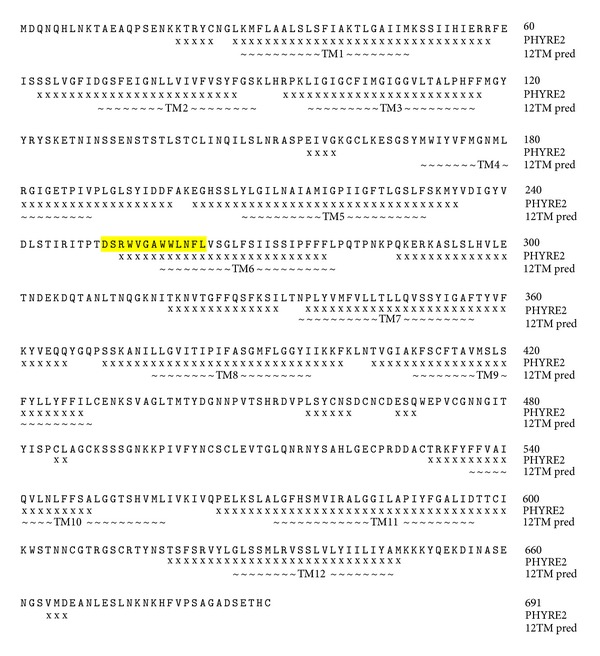
Regions of the OATP1B1 protein predicted to be *α*-helical by PHYRE2 (x) [40], and the average positions of the TMs as predicted by the algorithms that gave a 12-TM model (~). The signature sequence is highlighted in yellow.

**Figure 2:**
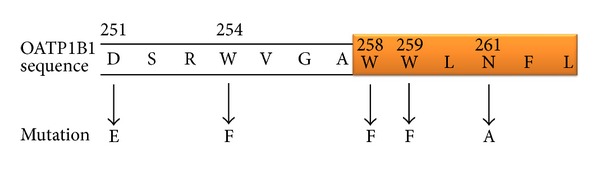
OATP1B1 signature sequence with amino acid positions and mutations performed in this study. The region of the sequence predicted to form part of TM 6 is highlighted in orange.

**Figure 3:**
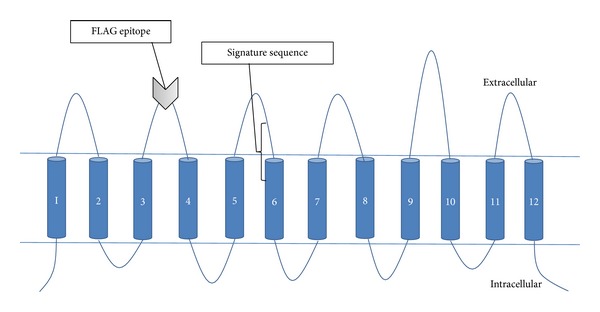
Predicted 12-TM topology of OATP1B1 with intracellular N and C termini. The FLAG epitope was inserted into the extracellular loop 2 between TMs 3 and 4 as directed by the topology predictions. The signature sequence is highlighted at the border between extracellular loop 3 and TM 6.

Homology modelling with the program PHYRE2 also gave a 12-TM prediction based on GlpT [41], against which 65% of the OATP1B1 residues were modeled with >90% confidence. The TM regions were consistent with those predicted by the topology prediction software (Figure 1). The software giving 12 TMs predicted the signature sequence to span the extracellular loop 3 from D251-A257 and TM 6 from W258-L263 (Figure 2). The combined topology results identified the putative extracellular loop between TMs 3 and 4 for the insertion of the FLAG epitope (Figure 3).

### 3.2. Functional Characterization of Wild Type OATP1B1 and Validation of OATP1B1-FLAG

**Figure 4:**
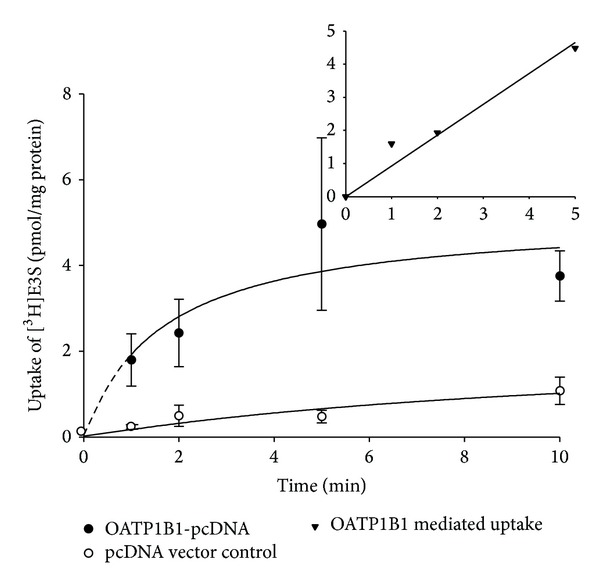
Uptake of 0.1 *μ*M [^3^H]E3S at 37°C between 0.5 and 10 min measured in HEK293T cells transiently transfected with OATP1B1-pcDNA (closed circles) or pcDNA vector control (open circles). Each point represents the mean ± standard error (SE) of 3-well replicates. Inset: OATP1B1-mediated uptake (i.e., OATP1B1-pcDNA uptake with the pcDNA vector control subtracted) up to 5 min, fitted with a linear regression line.

Transport function of OATP1B1 was quantified by measuring the uptake of the radiolabeled substrate [^3^H]E3S into OATP1B1 transiently transfected HEK293T cells. [^3^H]E3S uptake was linear up to 5 minutes (Figure 4, inset); therefore kinetic experiments were performed using a 3-minute uptake. Although biphasic kinetics is associated with OATP1B1 [44, 45], only the high affinity binding site was apparent under our experimental conditions (0.1 *μ*M [^3^H]E3S), with a *K*_*m*_ of 0.105 ± 0.008 *μ*M and *V*_max⁡_ of 50.1 ± 10.9 pmol/mg protein/3 min (Figure 6(a)), comparable to those found by other studies [46, 47].

### 3.3. Validation of the OATP1B1-FLAG Construct

**Figure 5:**
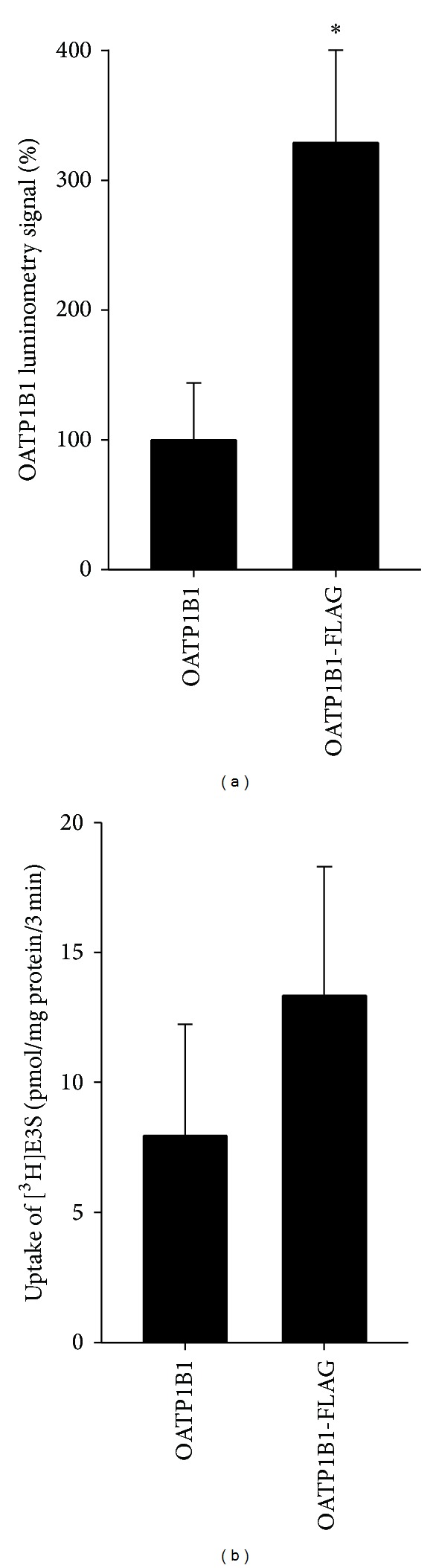
(a) Luminometry results from OATP1B1 and OATP1B1-FLAG transfected HEK293T cells. The OATP1B1-FLAG is expressed as a percentage of the OATP1B1 (no epitope) data. Each bar is the mean ± SE of 6 individual experiments with 3 dish replicates. *Statistical increase (*P* < 0.05) compared to OATP1B1 (no epitope) as determined by an unpaired *t*-test (Graphpad Prism). (b) Mediated uptake of 0.1 *μ*M [^3^H]E3S was measured at 37°C for 3 min into HEK293T cells transiently transfected with OATP1B1 and OATP1B1-FLAG constructs. Data are shown as uptake in pmol/mg protein/3 min with the pcDNA vector control uptake subtracted. Each bar represents the mean ± SE of 7 individual experiments with 6-well replicates. Uptake compared to vector only control was statistically significant (*P* < 0.05, unpaired *t*-test; Graphpad Prism).

Luminometry experiments were performed to validate the extracellularly located FLAG epitope on the OATP1B1 protein in nonpermeabilized cells. A significant increase in luminescence was observed in the OATP1B1-FLAG construct from the wild type OATP1B1 (Figure 5(a)), confirming that the FLAG epitope was placed in an extracellular portion of the protein.

**Figure 6:**
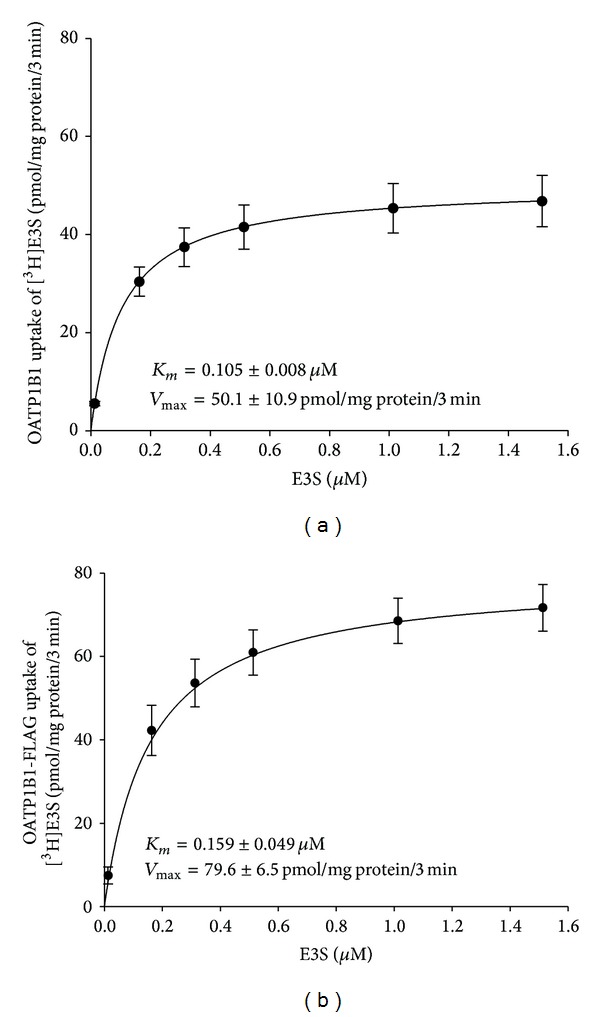
Uptake of increasing concentrations of 0.1 *μ*M [^3^H]E3S + 0–1.5 *μ*M E3S was measured at 37°C after 3 min from (a) OATP1B1 and (b) OATP1B1-FLAG transiently transfected HEK293T cells. The line is the Michaelis-Menten equation fitted to the *K*_*m*_ and *V*_max⁡_. Each point is the mean ± SE of 3 individual experiments with 3-well replicates and the pcDNA vector control uptake was subtracted.

Transport experiments with [^3^H]E3S were conducted to confirm that the OATP1B1-FLAG construct maintained function following the addition of the epitope. [^3^H]E3S uptake was measured alongside the OATP1B1 wild type (Figure 5(b)). The OATP1B1-FLAG construct retained transport function, with rates of uptake comparable to the untagged (no epitope) OATP1B1. Transport kinetics were performed with the OATP1B1-FLAG construct (Figure 6(b)), producing a *K*_*m*_ of 0.159 ± 0.049 *μ*M and *V*_max⁡_ of 79.6 ± 6.5 pmol/mg protein/3 min. There was no significant difference between the *K*_*m*_ values obtained for the FLAG-tagged construct and the wild type transporter. The *V*_max⁡_ increased compared to the wild type but not significantly.

### 3.4. Transport and Surface Expression of the OATP1B1-FLAG Signature Sequence Mutants

**Figure 7:**
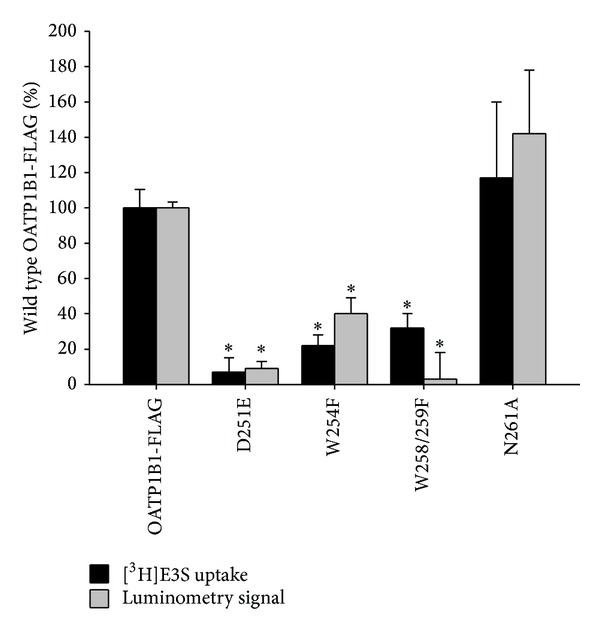
[^3^H]E3S uptake and luminometry results for OATP1B1-FLAG control and D251E, W254F, W258/259F, and N261A mutant constructs, transiently transfected into HEK293T cells. Uptake of 0.1 *μ*M [^3^H]E3S at 37°C after 3 min was measured and pcDNA vector control uptake was subtracted. The OATP1B1-FLAG [^3^H]E3S uptake was 15.4 pmol/mg protein/3 min. Luminometry experiments were measured as RLU/mg protein and OATP1B1 (no epitope) negative control values were subtracted. Mutant values were normalized to those of the OATP1B1-FLAG and expressed as a percentage. Each bar is the mean ± SE of 3 experiments (luminometry = 3 dish replicates, uptake = 6-well replicates). ∗Statistical decrease (*P* < 0.05) from the OATP1B1-FLAG was determined by a two-tailed one sample *t*-test (Sigmaplot 11.0).

Transport experiments were conducted to elucidate whether the OATP1B1-FLAG signature sequence mutants maintained function following site-directed mutagenesis. Uptake of [^3^H]E3S by the mutants was measured alongside the OATP1B1-FLAG construct (Figure 7).

Transport was significantly reduced from the OATP1B1-FLAG for D251E, W254F, and W258/259F mutants. There was no significant difference in transport for N261A, nor were the kinetic parameters changed from the OATP1B1-FLAG (N261A *K*_*m*_  0.084 ± 0.033 *μ*M, *V*_max⁡_  47.0 ± 19.4 pmol/mg protein/3 min). The low transport levels for the remaining mutants meant that kinetic data could not be accurately determined.

Luminometry was performed on the D251E, W254F, W258/259F, and N261A signature sequence mutants to quantitatively measure surface expression in comparison to the nonmutated OATP1B1-FLAG construct (Figure 7). Values were normalized to those of the OATP1B1-FLAG to facilitate the comparison. Surface expression was reduced significantly compared to the nonmutated OATP1B1-FLAG for D251E, W254F, and W258/259F. N261A surface expression was not significantly different from the OATP1B1-FLAG.

**Figure 8:**
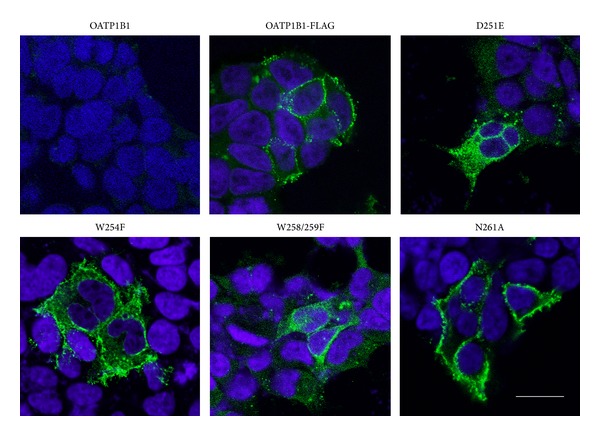
Immunofluorescence of OATP1B1 negative control, OATP1B1-FLAG positive control, D251E, W254F, W258/259F, and N261A transiently transfected HEK293T cells, visualized by confocal microscopy. DAPI (blue) localized to the nucleus (504 nm) and FITC (green) localized to the FLAG epitope (488 nm). Scale bar = 20 *μ*M.

Immunofluorescence experiments were performed to further investigate protein localization and expression. OATP1B1 expressing HEK293T cells were incubated with a primary anti-FLAG and secondary-FITC conjugated antibody. The nucleic acid stain DAPI was also used to visualize the cell nucleus.  Figure 8 shows the confocal microscopy images recorded from each mutant, for comparison with the OATP1B1-FLAG positive control and OATP1B1 negative control. The OATP1B1-FLAG control displayed predominantly membrane specific FITC fluorescence, as the N261A mutant did. For the remaining mutants, D251E, W254F, and W258/259F, the fluorescence was seen throughout the cell rather than at the membrane.

## 4. Discussion

OATP1B1 is one of the most influential members of the OATPs, owing to its role in the transport of many clinically relevant compounds into the hepatocyte. The transporter is central to the targeted drug delivery of statins [7], complicated by the presence of approximately 40 SNPs in the* SLCO1B1* gene sequence [5]. It is likely that along with other hepatic transporters and enzymes OATP1B1 plays an important part in the ADME of compounds. Therefore this particular transporter was apt for studying the structural characteristics responsible for protein function and surface expression.

OATP1B1 was predicted by 10 topology prediction programs and by GlpT homology modelling to contain 12 TMs with intracellular N and C termini. This information allowed the introduction of a FLAG epitope in extracellular loop 2 to facilitate the detection of surface expression. The 13 amino acid signature sequence is present in all OATPs and is highly conserved between species [4]. Its location at the interface between extracellular loop 3 and TM 6 suggests a structural role in protein folding and/or stability within the membrane. Unfortunately this region of OATP1B1 is not represented in the best PHYRE2 generated homology model, that with the crystal structure of GlpT as the template, so site-directed mutagenesis was used to investigate the role of conserved residues in the signature sequence.

The highly conserved amino acids which form the signature sequence of OATP1B1 were found to be important for surface expression. The introduction of D251E, W254F, and W258/259F to OATP1B1 significantly reduced surface expression and transport of [^3^H]E3S. Confocal microscopy showed that protein was in fact produced but was predominantly retained in the cytoplasm, suggesting a role of D251, W254, and W258/9 for protein folding and/or membrane localization. These amino acids therefore are likely to have a structural rather than functional role in the protein.

Tryptophan is a unique amino acid, exhibiting a large nonpolar surface area, the capability for hydrogen bond formation from the indole N–H moiety and the greatest electrostatic potential for noncovalent interactions [23]. Mutating tryptophan to phenylalanine dramatically reduced transport function, suggesting that the indole ring may be an important feature for hydrogen bonding and membrane stability in OATP1B1 [20, 23]. In a previous study, individual mutation of W258 and W259 to phenylalanine did not affect surface expression [21]. Our results therefore suggest that at least one tryptophan is required in this region of the protein, as functional expression is not affected upon single mutation of W258 or W259 to phenylalanine, but is abolished upon the double mutation. However the double tryptophans in rat OATP1C1 retained wild type characteristics when mutated to phenylalanine [22], suggesting that different OATPs may be able to function with the phenylalanine substitution.

The mutation of aspartic acid to glutamic acid (D251E) significantly reduced transport and surface expression. As both amino acids are negatively charged polar residues, it may be that the extra methylene group in glutamic acid disrupted protein folding. It has previously been reported that aspartic acid may interact with tryptophan more frequently than glutamic acid, following analysis of the spatial contacts and solvent accessibility of the amino acids [48]. Therefore it may also be that mutating to glutamic acid may have changed the interactions with tryptophan, disrupting the structure. D251E along with W254 was predicted to form part of the extracellular loop 3, a region of the signature structure which has not previously been studied. N261A was the only mutant to retain transport function and expression on the membrane, supported by a previous study [21], suggesting that the maintenance of a neutral charge is sufficient at this position.

## 5. Conclusions

The amino acids D251, W254, and W258/9 within the signature sequence of OATP1B1 are important for protein surface expression. Site-directed mutagenesis and topology predictions are powerful tools for analyzing protein topology and structural and functional roles of amino acids as currently no mammalian drug transporters have been crystallized [21]. These results will contribute to the growing database of structural and functional information on the OATPs and will inform their role in drug delivery.

## Figures and Tables

**Table 1:** The human OATPs with each signature sequence region. OATP1B1 amino acids mutated during this study are bold and underlined. Source: NCBI [18].

OATP	Signature sequence												
OATP1A2	D	T	R	W	V	G	A	W	W	F	G	F	L
**OATP1B1**	**D**	S	R	**W**	V	G	A	**W**	**W**	L	**N**	F	L
OATP1B3	D	S	R	W	V	G	A	W	W	L	G	F	L
OATP1C1	D	P	Q	W	V	G	A	W	W	L	G	Y	L
OATP2A1	D	P	R	W	I	G	A	W	W	L	G	L	L
OATP2B1	D	P	R	W	V	G	A	W	W	L	G	F	L
OATP3A1	D	P	R	W	I	G	A	W	W	G	G	F	L
OATP4A1	S	P	L	W	V	G	A	W	W	V	G	F	L
OATP4C1	D	P	R	W	L	G	A	W	W	I	G	F	L
OATP5A1	D	P	R	F	I	G	N	W	W	S	G	F	L
OATP6A1	S	P	E	W	L	W	T	W	W	I	N	F	L
